# Rosai-Dorfman disease in the central nervous system with two isolated lesions originated from a single clone: a case report

**DOI:** 10.1186/s12883-021-02379-2

**Published:** 2021-09-13

**Authors:** Huawei Jin, Zhenhua Yu, Tian Tian, Guoping Shen, Weian Chen, Miao Fan, Qun He, Fei Xu, Dawei Liu

**Affiliations:** 1 Department of Neurosurgery, the First Affiliated Hospital of Sun Yat-sen University, 58th, The Second Zhongshan Road, Guangzhou, Guangdong China; 2 Department of Pathology, the First Affiliated Hospital of Sun Yat-sen University, 58th, The Second Zhongshan Road, Guangzhou, Guangdong China; 3 Department of Radiation Oncology, the First Affiliated Hospital of Sun Yat-sen University, 58th, The Second Zhongshan Road, Guangdong Guangzhou, China; 4 Department of Nuclear Medicine, the First Affiliated Hospital of Sun Yat-sen University, 58th, The Second Zhongshan Road, Guangdong Guangzhou, China; 5 Department of Radiology, the First Affiliated Hospital of Sun Yat-sen University, 58th, The Second Zhongshan Road, Guangzhou, Guangdong China; 6 GenomiCare Biotechnology (Shanghai) Co. Ltd, No 111 Xiangke Road, Shanghai, China

**Keywords:** Central Nervous System, Clone origin, Mutation, Rosai–Dorfman disease

## Abstract

**Background:**

Rosai–Dorfman disease (RDD) is a rare, benign, idiopathic non-Langerhans cell histiocytosis. Cases of RDD in the CNS are extremely rare but lethal. RDD is thought to represent a reactive process. Recent studies proposed a subset of RDD cases that had a clonal nature. However, its clone origin is poorly understood.

**Case presentation:**

We present a rare case of RDD in the CNS with two isolated lesions. These two lesions were removed successively after two operations. No seizure nor recurrence appears to date (2 years follow-up). Morphological and immunohistochemical profiles of these two lesions support the diagnosis of RDD. Based on the whole-exome sequencing (WES) data, we found the larger lesion has a higher tumor mutational burden (TMB) and more driver gene mutations than the smaller lesion. We also found seven common truncal mutations in these two lesions, raising the possibility that they might stem from the same ancestor clone.

**Conclusions:**

Overall, this is the first report about clonal evolution of RDD in the CNS with two isolated lesions. Our findings contribute to the pathology of RDD, and support the notion that a subset of cases with RDD is a clonal histiocytic disorder driven by genetic alterations.

## Background

Rosai–Dorfman disease (RDD) is a rare, idiopathic non-Langerhans cell histiocytosis that affects children and young adults. Histologically, RDD is characterized by the histiocytic proliferation in lymph nodes or extranodal tissues. Immunohistochemical staining shows that lesional histiocytes are CD68^+^, S-100 ^+^, and CD1a ^-^, with prominent emperipolesis [1]. Cases involving the CNS are extremely rare, but they proved to be fatal due to the position [2]. A retrospective analysis showed that in the period between 1969, when the disease was first described, and 2014, only 210 cases of CNS involvement were reported [3]. RDD is considered as a reactive, nonclonal disorder. However, some evidences support a clonal nature of RDD, at least in a subset of cases [4, 5]. Still, the clone origin of RDD is poorly understood.

We identified a rare case of RDD isolated to CNS with two separate lesions. In order to understand the clone origin, we performed the whole-exome sequencing (WES) and made a comparison between two lesions based on sequencing data. We found the large neoplastic lesion had a higher TMB and more driver genes than the small lesion. Meanwhile, we also found seven common truncal mutations in two lesions, indicated that they might stem from the same ancestor clone. These findings provide new sight into the pathology of RDD and may introduce the option of targeted therapy.

## Case presentation

This study is approved by our institutional review board and this patient has signed informed consent.

**Fig. 1:**
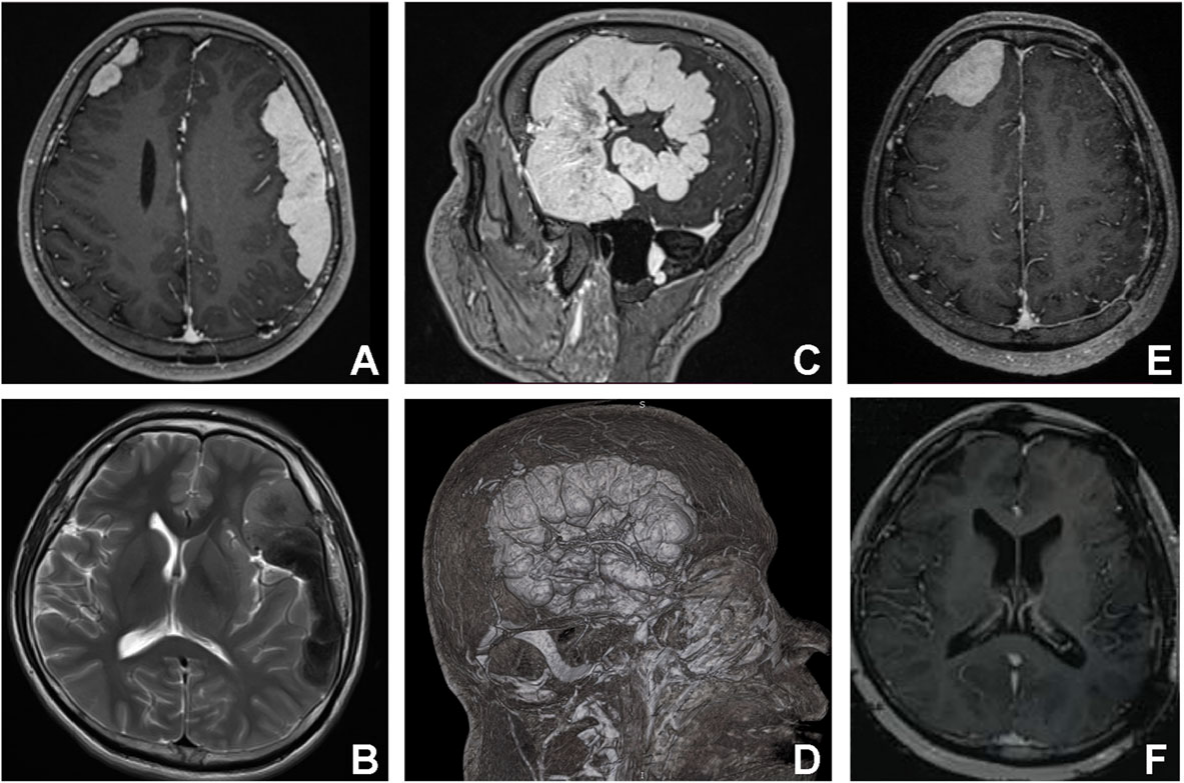
Radiological findings. **A.** Axial T1-weighted MRI before the first surgery demonstrates two extra-axial, homogeneous, well-defined, dural-based obviously uniform enhancement lesions, including a large lesion (93mm × 80mm **× **38mm) in the left frontotemporal parietal region, and a small lesion (38mm × 35mm × 11mm) in the right frontal region. **B.** Axial T2-weighted MRI before the first surgery. **C.** Sagittal T1-weighted MRI before the first surgery. **D.** 3D reconstruction from images before the first surgery shows the large lesion is rich blood supply which is mainly supplied by the middle meningeal artery. **E.** Axial T1-weighted MRI shows the right frontal lobe lesion grows obviously (50 mm **×** 50mm **×** 25 mm) after 20 months. **F.** Axial T1-weighted MRI shows that there is no tumor recurred in 12 months after the second surgery

A 33-year-old Chinese male initially presented with an epileptic seizure. Magnetic resonance imaging (MRI) detected two extra-axial, homogeneous, well-defined, dural-based lesions. The large one is in the left frontotemporal parietal region, and the small one is in the right frontal region. T1-weighted images show isointense and slightly hyperintense signal intensity, and T2-weighted images show isointense with intralesional hypointense foci, obviously uniform enhancement with an enhancing dural tail sign (Fig. 1). This patient was initially diagnosed with multiple convex meningiomas and underwent a craniotomy for resection of the large lesion. After the left lesion was resected, this patient recovered very well and restored normal speech function. We recommended adjuvant hormone therapy or radiotherapy but the patient declined any further treatment until progression was seen on the MRI and the patients suffered from a symptomatic seizure 20 months after surgery. MRI showed the size of the right lesion increased obviously. Then the second craniotomy was taken to remove the right lesion, and the patient recovered again with no neurological deficit. There was no seizure nor tumor recurrence on follow-up so far (2 years follow-up).

**Fig. 2:**
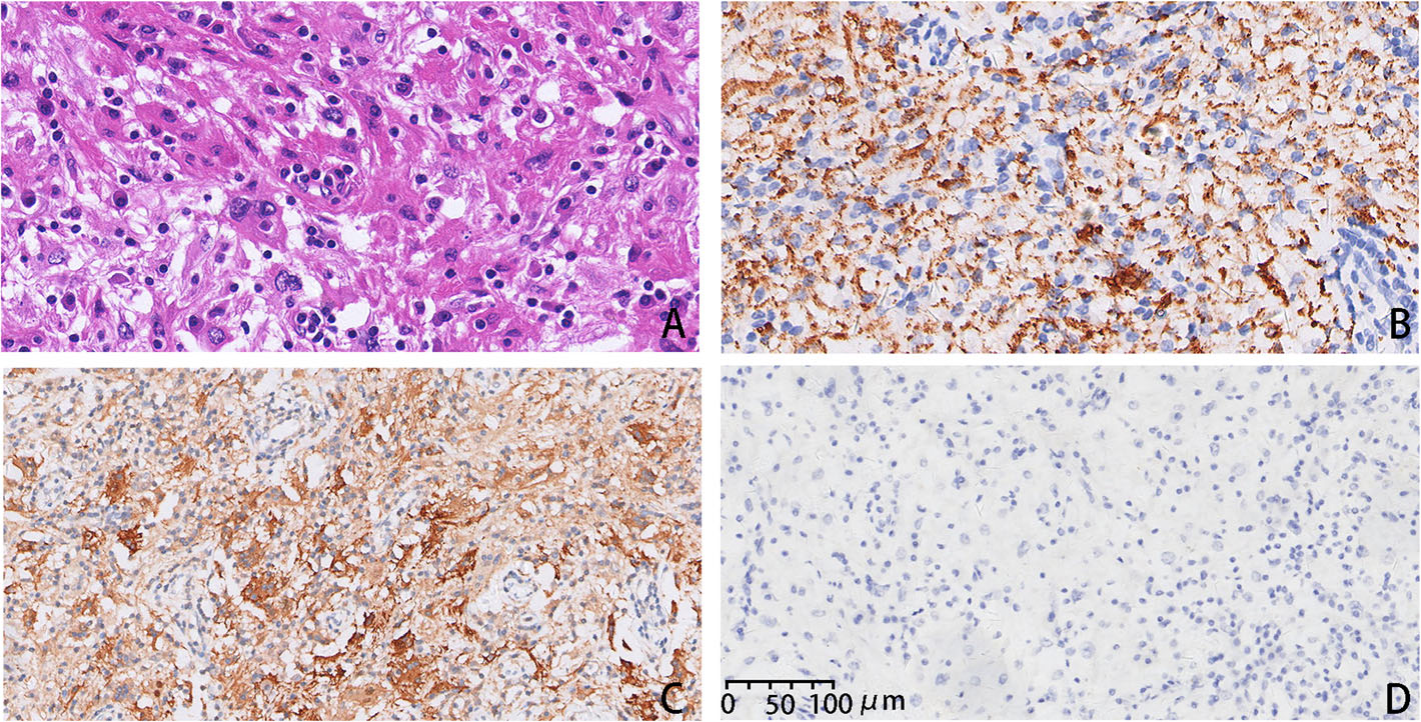
Histopathology of Rosai-Dorfman disease from the first operation. **A.** Large histiocytes with emperipolesis (HE, 40×). **B.** Histiocytes with emperipolesis, immunoreactive for CD68 (40×). **C.** Histiocytes with emperipolesis, immunoreactive for S-100 (40×). **D.** Histiocytes with emperipolesis, immunoreactive for CD1a (20×)

In the sections from the first operation, microscopic examination showed a large number of lymphocytes and plasma cells infiltrated in fibrous tissue scattered large histiocytic cells with abundant cytoplasm. Immunohistochemical staining showed positive for S100, CD68, but negative for CD1a (Fig. 2). Both morphological and immunohistochemical profiles of the left lesion supported the diagnosis of RDD.

**Fig. 3:**
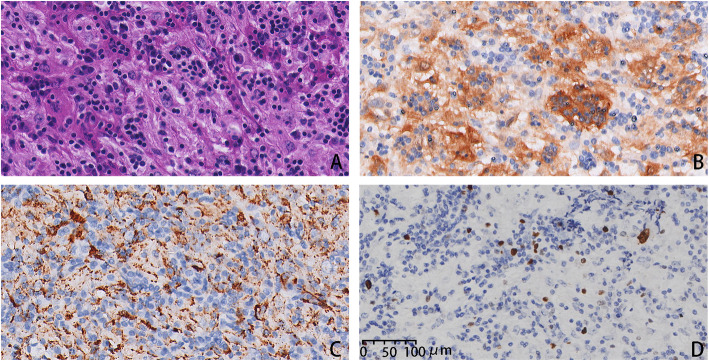
Histopathology of Rosai-Dorfman disease from the second operation. **A.** Histiocytes with emperipolesis (HE, 40×). **B.** Large histiocytes with emperipolesis, immunoreactive for S-100 (40×). **C.** Histiocytes with emperipolesis, immunoreactive for CD68 (40×). **D.** Histiocytes with low proliferation index, immunoreactive for Ki-67 (40×)

Histopathological examination for the sections from the second operation exhibited similar results as the first one (Fig. 3). Histiocytes were the predominant cells and contained abundant pale to vacuolated cytoplasm and mildly pleomorphic vesicular nuclei with multiple prominent nucleoli and emperipolesis.

**Fig. 4:**
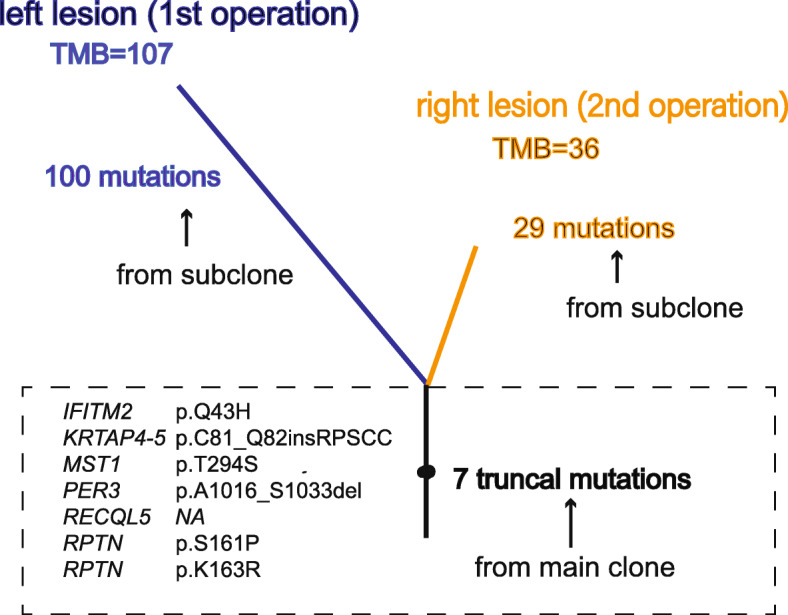
Evolution tree in two lesions of Rosai-Dorfman disease

Based on whole exome sequencing (WES) data, we calculated the tumor mutation burden (TMB), obtained driver genes, and performed clonality analysis. The larger (left) neoplastic lesion has a higher TMB (3.06/Mb) and more tumor-driven mutations than the smaller (right) one (1.03/Mb). Four driver gene mutations, including *PIK3R2*, *MED12*, *SUFU*, and *SOX2* were found in the larger lesion, while only one tumor-driven mutation (*NOTCH2*) was found in the smaller lesion. There are seven common truncal mutations in the two lesions (Fig. 4). None of them are driver gene mutations. These mutations rarely appear in other populations, indicating that these two lesions may originate from the same main ancestor clone.

## Discussion and conclusions

Historically, RDD has been considered as a reactive, non-clonal, non-neoplastic lesions, self-limited disorder of unknown etiology with few patients have poor outcomes. However, patients with RDD have been reported to undergo malignant transformation to histiocytic sarcoma [6] and high-grade lymphoma [7], which indicates their potential for genomic instability. Recent studies have found several mutations in lesional tissues [8, 9], raising the possibility of a clonal origin in some forms of RDD.

Nowadays, through deep-sequencing, multi-region sequencing, and single-cell sequencing, it is proved that almost every tumor evolution begins with a single normal cell, shares a set of truncal mutations in the normal tissue, transforms and expands to form a tumor mass [10]. If tumor subclones have no differential survival within a population, loss of positive selection would mean that the population evolves neutrally (only mutation and drift are at play). If tumor subclones gradually accumulate mutations and show tumor-driver alterations, they would evolve in a Darwinian fashion with clonal selection, favor the expansion of some lineages over others, lead to clonal lineages diverge, and slowly form distinct subpopulations of neoplastic lesions [11].

In this study, we described a rare case of CNS involvement with two isolated lesions. Based on WES data, we found seven common truncal mutations in two lesions, indicated that these two lesions might stem from the same main ancestor clone. These truncal mutations are all reported for the first time in RDD and none of them are tumor driver genes. Both *IFITM2* and *MST1* involve in immune response [12, 13]. *RECQL5* plays an important part in the maintenance of genome stability [14]. *PER3* is a circadian pathway gene and often reported to associate with tumor progression [15, 16]. For *KRTAP4-5* and *RPTN*, there is no direct evidence to support their role in tumorigenesis. In addition, we also found that the large neoplastic lesion had a higher TMB and more driver mutations than the small lesion. We retrieved previous CNS RDD studies and attempted to compare mutation landscape between our case and published data. However, limited data could be found in published studies. Only one case report showed *BRAF* mutation in a CNS RDD case by using targeted sequencing [17]. Other RDD studies revealed several mutated genes which mainly focused on MAPK/ERK pathway but lesions were not from CNS [4, 8]. These MAPK/ERK pathway related mutations were not found in our case, suggesting RDD might be a heterogeneous histiocytosis. Our data filled the gap in the mutational landscape of CNS RDD.

Our findings support the notion that RDD is a clonal proliferation of histiocytic disorder in a subset of patients. We believe that when no driver mutations emerge, histiocytes would evolve neutrally, show reactive proliferation, and grow more slowly, or even remit spontaneously. However, if driver mutations appeared under the accumulation of mutations, the lesion would evolve in a Darwinian fashion with clonal selection, growth acceleration, and lineages expansion. The more driver mutations gained, the faster the lesion grows. As in our case, the large lesion has a higher TMB and more driver mutations than the small one.

To the best of our knowledge, this is the first report about clonal evolution of RDD in the CNS with two isolated lesions. We confirmed that at least a subset of cases with RDD is a clonal histiocytic disorder driven by genetic alterations. Further genetic studies on RDD should be taken to better characterize its pathogenesis as well as open up potential avenues for therapy.

## Data Availability

We carefully documented the patient’s data reported in the article. We will share the de-identified data at the request of other qualified investigators for purposes of scientific research or teaching. To request the data, please contact the corresponding authors.

## Abbreviations

RDD: Rosai–Dorfman disease
CNS: Central nervous system
WES: Whole-exome sequencing
TMB: Tumor mutational burden
MRI: Magnetic resonance imaging
